# iPSC modification strategies to induce immune tolerance

**DOI:** 10.1093/lifemedi/lnaf016

**Published:** 2025-03-31

**Authors:** Zixuan Hong, Yun Zhao, Sara Pahlavan, Xue Wang, Sen Han, Xi Wang, Kai Wang

**Affiliations:** Department of Physiology and Pathophysiology, School of Basic Medical Sciences, State Key Laboratory of Vascular Homeostasis and Remodeling, Beijing Advanced Center of Cellular Homeostasis and Aging-Related Diseases, Clinical Stem Cell Research Center, Peking University Third Hospital, Peking University, Beijing 100191, China; Department of Physiology and Pathophysiology, School of Basic Medical Sciences, State Key Laboratory of Vascular Homeostasis and Remodeling, Beijing Advanced Center of Cellular Homeostasis and Aging-Related Diseases, Clinical Stem Cell Research Center, Peking University Third Hospital, Peking University, Beijing 100191, China; Department of Stem Cells and Developmental Biology, Cell Science Research Center, Royan Institute for Stem Cell Biology and Technology, ACECR, Tehran 16635-148, Iran; Department of Obstetrics and Gynecology, State Key Laboratory of Female Fertility Promotion, Peking University Third Hospital, Institute of Advanced Clinical Medicine, Peking University, Beijing 100191, China; Department of Thoracic Oncology II, Key Laboratory of Carcinogenesis and Translational Research (Ministry of Education), Peking University Cancer Hospital and Institute, Beijing 100142, China; Department of Physiology and Pathophysiology, School of Basic Medical Sciences, State Key Laboratory of Vascular Homeostasis and Remodeling, Beijing Advanced Center of Cellular Homeostasis and Aging-Related Diseases, Clinical Stem Cell Research Center, Peking University Third Hospital, Peking University, Beijing 100191, China; Department of Obstetrics and Gynecology, State Key Laboratory of Female Fertility Promotion, Peking University Third Hospital, Institute of Advanced Clinical Medicine, Peking University, Beijing 100191, China; Department of Physiology and Pathophysiology, School of Basic Medical Sciences, State Key Laboratory of Vascular Homeostasis and Remodeling, Beijing Advanced Center of Cellular Homeostasis and Aging-Related Diseases, Clinical Stem Cell Research Center, Peking University Third Hospital, Peking University, Beijing 100191, China

**Keywords:** hPSC, HLA, gene editing, immune checkpoint

## Abstract

Human pluripotent stem cells (hPSCs) hold great promise in regenerative medicine. However, immune rejections remain one of the major obstacles to stem cell therapy. Though conventional immunosuppressants are available in clinics, the side effects prevent the wide application of hPSCs derivatives, compromising both survival rate and quality of life. In recent years, a myriad of strategies aimed at inducing immune tolerance specifically by engineering stem cells has been introduced to society. One strategy involves human leukocyte antigen (HLA) deletion through gene editing, affording allografts the capability to evade the host immune system. Another strategy involves immune cloak, which is the focus of this review, with emphasis on the overexpression of immune checkpoints and the blocking of immune cytotoxic pathways. Nevertheless, co-transplantation with mesenchymal stem cells (MSCs) and enhanced MSCs confers immune privilege to engraftments. This review summarizes recent studies on the intricacies of immune tolerance induction by engineering stem cells. In addition, we endeavor to deliberate upon the safety and limitations associated with this promising and potential therapeutic modality.

## Introduction

Induced pluripotent stem cell (iPSC) unprecedentedly promotes the development of regenerative medicine. With the introduction of Oct3/4, Sox2, Klf4, and c-Myc, somatic cells can be reprogrammed into pluripotent stem cells and obtain the ability to differentiate into all cell types like the embryonic stem cell (ESC) [1]. The emergence of pluripotent stem cells (both ESC and iPSC) not only provides a powerful tool for research in cell fate determination and organ development but also offers personalized “seeds” to treat incurable degenerative diseases [2]. Patients with organ failure suffer physical pain and spiritual pressure waiting for an organ transplantation surgery. Subsequent difficulties include immune rejection in all likelihood and lifetime medication of immunosuppressants. All these realistic needs push forward the investigation of PSC-related clinical trials, but the major obstacle remains in ensuring the immune tolerance of PSC-derived allografts [3].

The immune system identifies nonself by recognizing the major histocompatibility complex (MHC). Class I MHC molecules, encoded by three different genes in 6p21.3 (*HLA-A*, *-B*, and *-C*), expressed in all nucleated cells, and presented intracellular peptides to CD8^+^ T cells. The α1 and α2 domains in the heavy chain contribute to the polymorphism of MHC-I, while the α3 domain is associated with the invariant light chain β-2-microglobulin. MHC class I-like genes (*HLA-E*, *-F*, and *-G*) share similar sequences with MHC-I but they play distinct functions and inhibit the host immune system. MHC-II molecules (HLA-DP, -DQ, and -DR) mainly exist in antigen-presenting cells including macrophage and dendritic cells, presenting processed extracellular antigens to CD4^+^ T helper cells [4]. Discrepant MHC, notably class I, can be recognized as an alloantigen and triggers the activation of an immune attack [5, 6].

Theoretically, iPSC owns identical MHC with its original donor, and thus should not be immune-excluded. In 2011, Zhao et al. first reported immune rejection in B6 mice to their iPSC-derived teratomas [7]. The phenomenon can be explained by different immunogenicity exhibited in iPSC and its derivatives due to the reprogramming and differentiation process [8, 9]. The controversy of graft versus host disease (GVHD) casts a shadow on stem cell therapy and ignites further researches in facilitating immunocompatibility.

**Figure 1.**
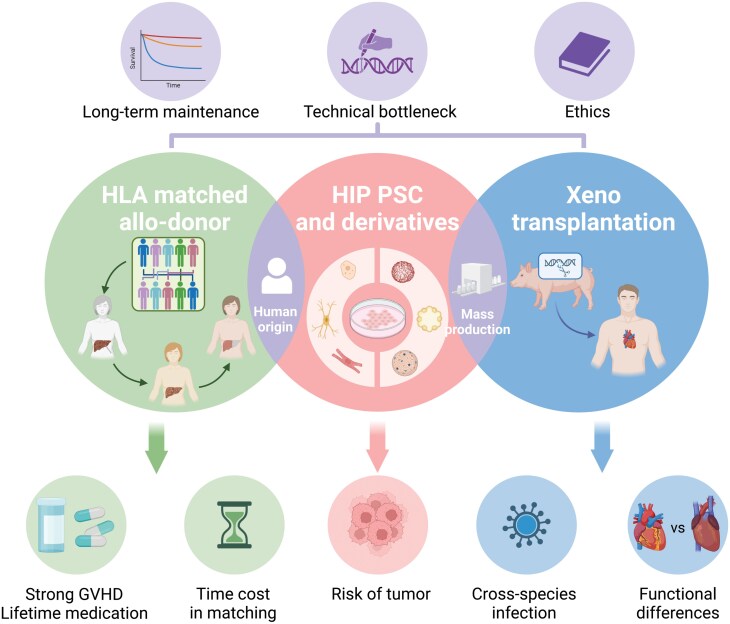
Comparison of three strategies and related issues in rescuing organ failure.

As with the standard solution after organ transplantation, current principles to overcome immune rejection include HLA matching and immune suppressive therapy. Waiting for a matching donor can be a drawn-out process, not to mention the countless side effects of the immunosuppressants [10, 11]. In terms of offering off-the-shelf stem cell therapy, several countries have set up HLA bank to store cell lines from HLA homozygous individuals [12]. Despite the substantial costs of maintaining the institution, the current stock is still inadequate to cover all rare types [3]. Besides, researchers are making great efforts to make xeno-transplantation come true, which represents another major sect in addressing the issue of donor scarcity [13]. Yet this places higher demands on the genetic editing of xenografts to reach immune tolerance. Considering the pros and cons of current means, an optimal strategy is modified hypoimmunogenic iPSC, which offers the best HLA matching and minimal side effects (Fig. 1).

**Figure 2.**
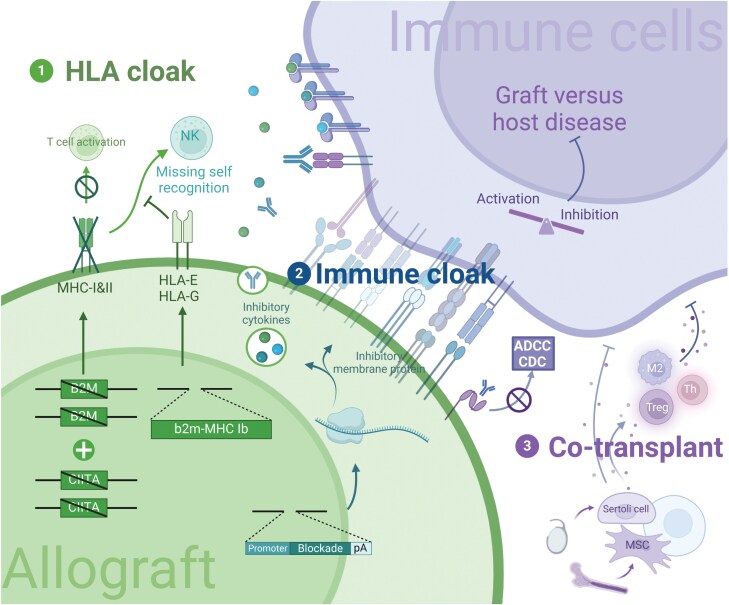
Strategies and mechanisms for establishing immune privileged hPSC.

Here, we provide an overview of three approaches in hPSCs modification in order to reduce immune rejection (Fig. 2). As for the HLA cloak, the classical scheme of MHC-I and MHC-II knock-out seems to be insufficient [14]. Thus novel strategies like selective deletion or B2M/HLA-E transgenes are preferred [15, 16]. Immune cloak is the star tactic nowadays. Researchers utilized multiple natural immune blockades like PD-L1, CD200, and CD47 [17, 18]. Synthetic engagers including truncated CD64 and LILRB1 engagers avoid activating complex functions like natural ones [19, 20]. Intervening immune-related pathways like IFN-γ signaling can prevent cytokine toxicity [21]. Another effective strategy is co-transplantation with protective cells [22, 23]. The ultimate goal is to induce long-term, antigen-specific immunologic unresponsiveness in a safe and secure manner.

## HLA cloak

The manipulation of MHC molecules sets the base for generating universal stem cells. In addition, tumor cells also undergo immune escape by downregulating MHC [24]. Double knock-out of B2M and CIITA, respectively, encoding β-2-microglobulin and class II major histocompatibility complex transactivator, has been widely adopted to overcome histoincompatibility. But loss of MHC activates “missing-self recognition” from natural killer (NK) cells which leads to lysis of transplanted cells [25]. Correspondingly, modified plans including selective deletion or introducing HLA-E/B2M transgene emerge to inhibit lysis (Table 1) [14–16, 26–29]. Since the advent of HLA modification, debate on safety issues simultaneously begins [30, 31]. Nevertheless, the HLA cloak is the undoubted foundation of universal stem cells.

**Table 1.** HLA manipulation strategies in hPSC.

Number	Cell type	HLA knock out			HLA knock in	Results	Reference
		HLA-Ia	HLA-Ib	HLA-II			
1	hESC	B2M^−/−^	B2M^−/−^	−	−	Evading CD8^+^ T cell but NK activation was not considered in SCID model	[26]
2	hiPSC	B2M^−/−^	B2M^−/−^	CIITA^−/−^	−	Derived cardiomyocytes displayed better contractile regularity after co-culture with human PBMC	[27]
3	hiPSC	B2M^−/−^	B2M^−/−-^	−	B2M~HLA-A*11:01^+^	Derived endothelial cells less induced CD8^+^ T cell and NK cell with HLA-A*11:01	[28]
4	hiPSC	HLA-A^−/−^ HLA-B^−/−^	−	CIITA^−/−^	−	HLA-C retained iPSC is more efficient in evading NK than HLA-C^−/−^ HLA-E^+^	[14]
		HLA-A^+/−^ HLA-B^+/−^ HLA-C^+/−^	−	−	−	Pseudo-homozygous hiPSC can evade T cell	
5	hESC	HLA-A^−/−^ HLA-B^−/−^ HLA-C^−/−^	−	CIITA^−/−^	HLA-A2^+^	Differentiated β-like cells evaded from T and NK cytotoxicity in 8 weeks	[16]
6	hESC	B2M^−/−^	B2M^−/−^	−	B2M~HLA-E^+^	HLA-E knock-in rescued B2M KO cell from NKG2A^+^ NK cell population	[15]
7	hESC	HLA-A^−/−^ HLA-B^−/−^ HLA-C^−/−^	−	CIITA^−/−^	HLA-G^+^ (PD-L1^+^ CD47^+^)	This triple knock-in cell line and its derivatives performed better than PD-L1^+^ CD47^+^ cell both *in vitro* and *in vivo*, indicating the vital role of HLA-G	[29]

### HLA knock-out

B2M locates in 15q21.1 and encodes β-2-microglobulin, the light chain of class I MHC. As a replacement for MHC matching, B2M knock-out has been widely adopted in xenograft and allograft transplantation to mitigate immune rejection. Due to the increasing susceptibility of NK, B2M knock-out is usually not applied as a separate strategy. Wang et al. described their work in B2M^−/−^ human ESC. They drew the conclusion that B2M KO minimized immunogenicity due to evading CD8^+^ T cells. Yet the activation of NK due to HLA-I insufficiency was not considered in their SCID mouse model [26]. Song et al. generated B2M^−/−^ iPSC but it increased NK cell cytotoxicity as described above. After transduction of HLA-A linked with B2M, the B2M KO cell line reduced immunogenicity compared to the wild type when co-culturing with human peripheral blood mononuclear cell (PBMC) and NK with identical HLA-A [28].

Double knock-out of classes I and II is essential when expression of MHC-II should be considered due to cell lineage features or experimental needs. The transcription of MHC-II acquires four essential transactivators, CIITA, RFX5, RFXANK, and RFXAP [5, 32]. Most researches targeted CIITA to silence MHC-II [33]. A representative work from Mattapally et al. reported the generation of B2M^−/−^CIITA^−/−^ iPS cell line via CRISPR/Cas9, which then was successfully differentiated into cardiomyocyte. They also observed decreased expression of HLA-E and HLA-F, but not the HLA-G after co-culture with PBMC. This phenomenon indicated one side effect of CIITA KO that it restrained inhibitory MHC-Ib [27].

Double knock-out (DKO) strategy disables the presentation of antigens from MHC to T cell receptors. Yet it is inadequate to maintain allograft cells unless combined with effective NK inhibition. Another limitation is the faint immune function and high risk of infection due to failure in presenting antigens [31]. In spite of the potential risks in the application, the emergence of B2M and CIITA KO sets the foundation of immune tolerance and attracts attention to HLA engineering.

### Amended HLA modification strategies to evade NK cell

Like other immune cells, NK cells are educated when encountering MHC-I-expressing cells in peripheral blood [34]. The lysis function is usually silent due to the reaction between MHC-I in all nucleated cells and inhibitory receptors on the surface of NK cells. CD94/NKG2A, B, and C bind HLA-E trimer consisting of HLA α chain, β2m, and a signal sequence peptide [4, 15]. Another category consists of inhibitory killer cell immunoglobulin-like receptors (KIRs) encoded by group A KIR gene family. HLA-C plays a pivotal role in NK cell inhibition by activating KIR2D [35].

According to the aforementioned mechanisms, recent studies focused on modifying MHC-I, selective deletion of MHC-Ia, or overexpression of MHC-Ib [3]. Xu et al. formulated two plans for HLA genes knock-out. They first explored preserving haplotype HLA-A, -B, and -C in iPSC from HLA heterozygous donors. The other preserved only haplotype HLA-C, with the exclusion of HLA-A and HLA-B biallelically. Both two strategies were competent in suppressing T and NK cell activity while retaining HLA expression [14]. In addition, it was estimated that 12 lines of HLA-C-retained MHC-II KO iPSCs are immunologically compatible with more than 90% of the world’s population, which significantly trims the expenses on the construction of library [3]. Transduction of HLA linked with β2m coding sequence in B2M^−/−^ cell line ensures recovery of unitary type of MHC molecules. Parent et al. retained HLA-A2 in B2M^−/−^ hESC. Differentiated β-like cells showed an increased survival rate in 8 weeks post-transplantation, indicating efficacious protection from T-cell-mediated killing and reduction of NK cell lysis [16].

HLA-E/B2M fusion protein to DKO iPSCs takes effect through combining inhibitory CD94/NKG2A [34]. Crew et al. generated an engineered HLA-E single-chain trimer which successfully protected porcine epithelial cells from human NK lysis [36]. A similar tech was applied in a work by Gornalusse et al. to knock in the fused gene by AAV-mediated gene editing [15]. Jo et al. introduced HLA-E/B2M to B2M loci to generate universal CAR T-cells [37]. HLA-G becomes a novel target due to its vital role in maternal–fetal interface. It was testified that forced expression of HLA-G led to NK inhibition [29, 38].

## Immune cloak

**Figure 3.**
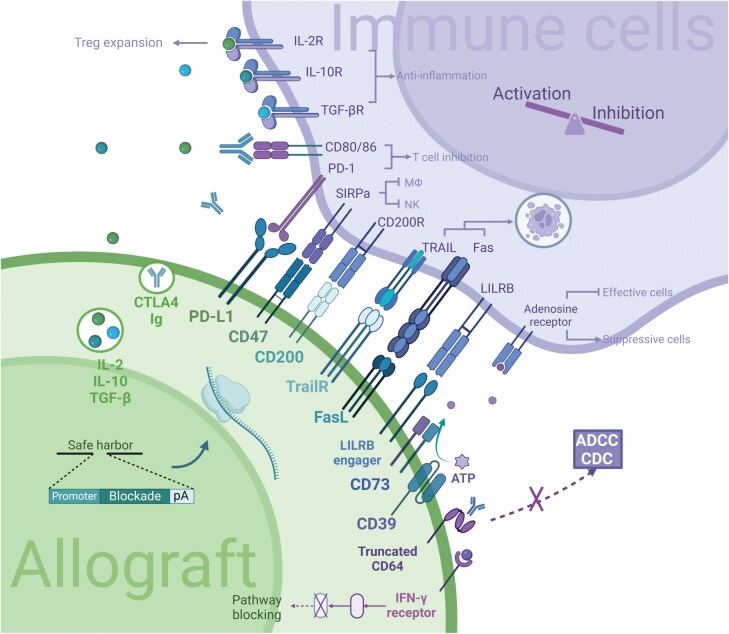
Immune inhibitory factors used in generating universal PSC.

HLA cloak strategy meets its Achilles’ Heel when it comes to certain lineages such as hematopoietic cells that constitute the immune barrier and require the integrity of MHC molecules [31]. In addition, minor histocompatibility antigens also lead to long-term GVHD [39]. To make up for these deficiencies, an immune cloak presents irreplaceable advantages in diminishing immune rejection. With the deepening understanding of immune mechanisms, soaring potential hits have been discovered and experimentally validated. However, it is still worth discussing how to achieve the best results with the simplest solution rather than piling up everything (Fig. 3).

### Natural immune checkpoint inhibitors

Natural immune checkpoints gain attention partially due to the requirement of cancer treatment. Tumor immunotherapy utilizes an immune checkpoint inhibitor (ICI), on the contrary, induction of immune tolerance focuses on the activator. A typical example is programmed cell death ligand 1 (PD-L1). Its receptor, programmed cell death protein 1 (PD-1), is often expressed during T cell activation to limit immune overreaction [40]. Yoshihara et al. used PD-L1 as the protection for human islet-like organoid derived from iPSC [41].

CD47 acts as the ligand of SIRPα to inhibit macrophages. It is now widely applied to inhibit NK cells [42]. Deuse et al. generated B2M^−/−^CIITA^−/−^CD47^+^ miPSC and hiPSC. Modified cells as well as derived endothelial cells and cardiomyocytes showed significantly prolonged survival after transplantation to humanized mice. Immune tolerance was also proved when modified miPSC and derivatives were transplanted to immunocompetent and immunodeficient mice [18]. Schrepfer and colleagues applied a similar strategy and differentiated HIP iPS to pancreatic β cells [43]. Based on the HLA-deficient engineering strategy, Han et al. knocked in PD-L1, HLA-G, and CD47 in the adeno-associated virus integration site 1 (AAVS1) locus. Multiple edited KI-PSC exhibited normal stemness and differentiation ability. *In vitro* coculture experiments concluded that KI-PSC-derived vascular smooth muscle cells and endothelial cells intrigued blunted T cell responses while minimizing NK and macrophage activity [29].

Several secretion cytokines including IL-10 and TGF-β may regulate the immune microenvironment to assist immune evasion. In a work presented by Melton and colleagues, they demonstrated that HLA deficiency and overexpression of PD-L1 were not enough to protect human-SC β cells. Secreting IL-2, IL-10, and TGF-β from the GAPDH locus of graft cells recruited CD8^+^ Foxp3^+^ regulatory T cells to inhibit immune attack. Engineered SC-islet cells could survive from xeno-rejection and reverse diabetes for 2–3 weeks [44]. Ward et al. made use of low-dose IL-2 to recruit regulatory T cell (Treg) and introduced a mouse IL-2/IL-2Rα fusion protein. With its high activity *in vivo*, the fused protein presented great clinical potential in inhibiting overactive immune response [45].

Although challenging, achieving immune evasion can also be accomplished without HLA editing and relying solely on the overexpression of inhibitory molecules. Harding et al. overexpressed eight immunomodulatory transgenes (*Pdl1*, *Cd200*, *Cd47*, *H2-M3*, *Fasl*, *Serpinb9*, *Ccl21*, and *Mfge8*) with FailSafe transgene system in mouse ESC to gain immune privilege after transplantation to outbred and allogeneic inbred recipients. Their human orthologues were then proved effective in abolishing the PBMC immune response [46]. Preservation of MHC molecules improved safety if infection is considered. Inducible expression systems such as TetOn allow the purgation of transplanted cells when necessary.

### Synthetic or modified blockers

Based on natural immune inhibitory molecules, researchers have crafted numerous synthetic or modified immunosuppressants through advanced techniques such as truncation, fusion, and majorization. CTLA4-Ig is a soluble fusion protein of the CTLA-4 variable region and human IgG1 constant region. In 1992, Lenschow et al. synthesized CTLA4Ig that bound both murine and human B7 to induce T cell unresponsiveness. The fusion protein showed 20-fold greater affinity than the natural receptor CD28 [47]. Expression of PD-L1 and CTLA4-Ig has since become a classic solution for combating immune rejection. Rong et al. introduced the PD-L1 and CTLA4-Ig into the HPRT1 locus of hESC. These engineered cells were successfully derived into fibroblasts and cardiomyocytes and showed low immune rejection after being transplanted into humanized mice [17]. According to Malek and colleagues, low dose IL-2 expanded Treg but not Teff rather than high dose. Thus they generated mouse IL-2/IL-2R fused protein presented longer persistency and higher activity *in vivo* [45].

The production of synthetic engagers relies on the fundamental principle of antigen–antibody binding, followed by screening to evaluate their physiological effects. For instance, Gravina et al. generated five engagers (respectively combining SIRPα, LILRB1, LILRB3, PD-1, and TIM3) among which the SIRPα engager avoids the natural function of CD47 in Ca^2+^ signaling, cell adhesion and migration. B2M^−/−^CIITA^−/−^SIRPα-E^+^-truncated CD64^+^ iEC were fully protected from NK lysis, antibody-dependent cellular cytotoxicity (ADCC), and complement-dependent cytotoxicity (CDC) [20]. While their work enlightens us to make artificial engagers, the correlated clinical trials must be taken into account to defuse all potential side effects.

CD64 (FcγRI) acts as an IgG Fc receptor to restrain ADCC and CDC. Deuse and colleagues assessed the survival of B2M^−/−^CIITA^−/−^CD47^+^tCD64^+^ endothelial cells *in vitro* and *in vivo*. Truncated or full-length CD64 effectively protected these hipo-immune iEC when co-culturing with macrophage (the effector cell of ADCC) and antibodies to intrigue the reaction. In NSG mice injected with immune cells and antibodies, the luciferase signal from transplanted iEC lasted up to 8 weeks [19].

### Intervening signal pathways

Forced expression of inhibitory molecules directly competes with the activation signals during the recognition and killing process. Intervening signal pathways to erode immune cells while stiffening transplanted cells can definitely help the allografts win this battle. Yet it inevitably requires detriments to the immune system. The vital role of apoptosis in peripheral immune tolerance has been discussed in both immune system development and conversion, which has inspired researchers to apply this strategy in transplant protection [48–50]. The classic celebrity pathway Fas and FasL has received widespread and sustained attention due to its crucial role in tumor immunotherapy. Forced expression of FasL on transplanted cells induced the apoptosis of immune cells by binding surface Fas [46]. Headen et al. co-transplanted allogeneic islets with streptavidin (SA)-FasL-presenting microgels. This was proved efficient in mice and monkeys [51–53]. Similarly, TRAILR (tumor necrosis factor-related apoptosis-inducing ligand receptor) is also practical. As a tumor suppressor, TRAIL induces tumor cell apoptosis through binding to TRAILR, including DR4 and KILLER/DR5 [54, 55].

The transporter associated with antigen processing (TAP) belongs to the ATP-binding cassette transporter family and is often considered a target in tumor formation and viral infection [56]. TAP knock-out ensures retaining MHC molecules while inhibiting antigen presentation. Clinical evidence has revealed that TAP1 or TAP2 mutation patients could be healthy while with a low level of class I HLA, which strongly contributes to the potential of this plan [32]. As a more moderate strategy, CD39 and CD73 ectonucleotidase receptors can hydrolyze ATP to generate pericellular adenosine, thereby downregulating Teffs and their proliferation [57].

Compared to damaging the immune system, strengthening transplanted cells is a better choice. Sintov et al. identified CXCL-10 as a target of immune evasion by CRISPR screening. Depleting CXCL-10 in stem-cell-derived islets may help to manipulate the IFN-γ pathway [21]. CRISPR screen is a powerful tool to achieve high throughput identification of functional genes involved in cellular processes. There are still many unexplored opportunities in harnessing the potential of CRISPR screen for immune evasion-related signal pathways, representing broad spaces for future investigation.

## Co-transplantation

Except for engineering simply on the allotransplant cell, another possible solution is to co-transplant with other cells guarding them from host immune attack. Some got inspiration from the natural blood-organ barrier and utilized Sertoli cells from the testes. In autoimmune diseases, mesenchymal stem cell (MSC) and Treg therapies hold great promise in impairing immune cells. Recent studies focus on engineering strategies to strengthen their maintenance and function, including forced cytokines expression and chimeric antigen receptor Treg (CAR Treg) [58, 59]. Another possible solution is to build a chimeric immune system by PSC–HSC co-transplantation. These companion cells can be developed to on-shelf products and used in autoimmune diseases and GVHD.

### Sertoli cell

Sertoli cells form a blood seminiferous barrier, dividing the testes into basal and epithelial regions. This barrier prevents harmful substances in the blood from entering the area of sperm formation and development, while allowing beneficial nutrients to pass through. They also excelled in secreting growth factors and immunomodulatory molecules [60]. Assiduous efforts were made to explore the immune protection from Sertoli cells. In 1993, Selawry and Cameron reported their work in co-transplantation of β cells and Sertoli cells to diabetic rats [61]. Xenotransplantation from rats to mice was also tested [62, 63]. Luca et al. established a protocol for the isolation and microencapsulation of porcine Sertoli cells compatible with long-haul transportation and could be potentially employable for xenotransplantation [64]. Yet it remains a defect that Sertoli cells might not be used in female recipients.

### Mesenchymal stem cell

MSC plays a vital role in tissue repair and has become a cutting-edge cell therapy for its broad anti-inflammatory talent [65]. In the work from Yoshida et al., simultaneous transplantation with syngeneic MSCs increased the expression of TGF-β and IL-2. Besides, they also discovered the induction of Treg and accelerating apoptosis of CD8^+^ cytotoxic T cells [66, 67]. Immune modulation on MSC may also contribute to the allograft survival. In a work from Wang et al., engineering MSC with expression of PD-L1 and CTLA4-Ig reduced the immune reaction in islet transplantation to T1D mice [23]. To improve the persistence of MSCs, other strategies such as priming MSCs with hypoxia, inflammatory cytokines, and small molecules were testified [65, 68, 69]. The prospects are generally promising as long as we establish a safe and reliable MSC production protocol. Quality control remains to be a nonnegligible question.

### Treg

As a subset of CD4^+^ T cells, Treg are characterized by high expression of IL-2 receptor CD25 and low expression of IL-7 receptor CD127 [70]. Treg-based cell therapies have been applied to GVHD patients to alleviate immune responses and to reduce the need for immunosuppression [71]. However, the maintenance of both population and function remains to be a question. Low-dose IL-2 stimulation is applied to induce Treg *in vivo* expansion. CAR Treg targets specific molecules to avoid broad immune suppression [59]. MacDonald et al. transplanted HLA-A2^+^ T cells into NSG mice to generate a xenogeneic GVHD model. Compared to polyclonal Treg, HLA-A2-CAR Treg performed better in inhibiting immune response [72]. In other studies, human CD19 CAR Treg has also proved effective in depleting B cells without triggering cytokine release syndrome [73].

### PSC–HSC co-transplantation

Induction of mixed chimerism expects to reach immune tolerance by compromising a mixture of host and donor hematopoietic stem cells (HSCs) [74]. Transplanting HSC with mismatched MHC was proved realistic, holding greater promise for the co-transplantation plan. Host pre-treatment with six monoclonal antibodies targeting CD47, T cells, NK cells, and HSCs followed by donor HSC transplantation [75]. However, the ethical controversy of this scenario is more serious. It needs to be considered whether this strategy could potentially lead to an unwarranted increase in unnecessary surgeries and inflict greater suffering on patients.

## Limitation

Though appealing, immune-tolerant stem cell still faces a bunch of obstacles on its way to fruition, including safety, quality, financial, and temporal limitations. These factors need to be taken into account and ethical and legal standards strictly adhered to in order to ensure safety and efficacy.

### Safety issue

Utilizing natural immune blockades cannot avoid activating unintended functions. This might be potentially addressed by synthetic engagers. Gravina et al. generated SIRPα engagers to replace CD47 and avoid its physiologic function in Ca^2+^ signaling and cell adhesion [20, 76]. Given that the binding of CD64 and IgG Fc may activate downstream signaling, truncated CD64 without the intracellular tail presents an equivalent function in preventing ADCC and CDC as its full-length product [19].

It is also crucial to suppress tumorgenicity. The infinite proliferation of PSC is a double-edged sword, yet the expression of immune inhibitory molecules sharpens both two edges [3, 77]. One major concern for regenerative medicine is the formation of tumors, especially teratomas. In the hipo-immune stem cells, manipulation of immunological surveillance echoes the evasion of tumor cells [24]. The key is to dislodge the undifferentiated cell and to suppress the dedifferentiation [78]. Liang et al. proposed the feasibility of linking a suicide gene to prevent tumorigenicity and viral infection [79, 80]

More are concerning whether immune-tolerant PSCs are safe to resist the pandemic or if it would lead to sturdy infection. Intact immune response against foreign pathogens requires the operation of antigen-presenting process. Particularly for the HLA cloak strategy, the disruption of normal immune response requires us to make a trade-off between fighting against GVHD and avoiding long-term infection [31, 81]. Even worse, infectious tumor transferring among allogenic individuals may become a truth like canine transmissible venereal tumor in dogs [30]. These foreseeing hazards need to be re-examined.

### Barriers in inducing immune tolerance

Current strategies of immune tolerance induction rely on genome editing, yet the safety of this tech itself remains to be testified. Off-target mutation has long been questioned [82]. Till now, we lack a robust expression system of immune modulatory factors that are not affected by the differentiation of PSC. Most researches utilized lentivirus to deliver immune blockade sequences, which raises the concerns that random inversion may interrupt normal genome and cause severe results [83]. These biosafety risks warrant close supervision. We need basic researches and clinical trials, including selecting appropriate gene editing techniques, conducting comprehensive evaluations of cell characteristics and functions, and long-term safety monitoring to ensure the safety and effectiveness of cell therapy.

**Figure 4.**
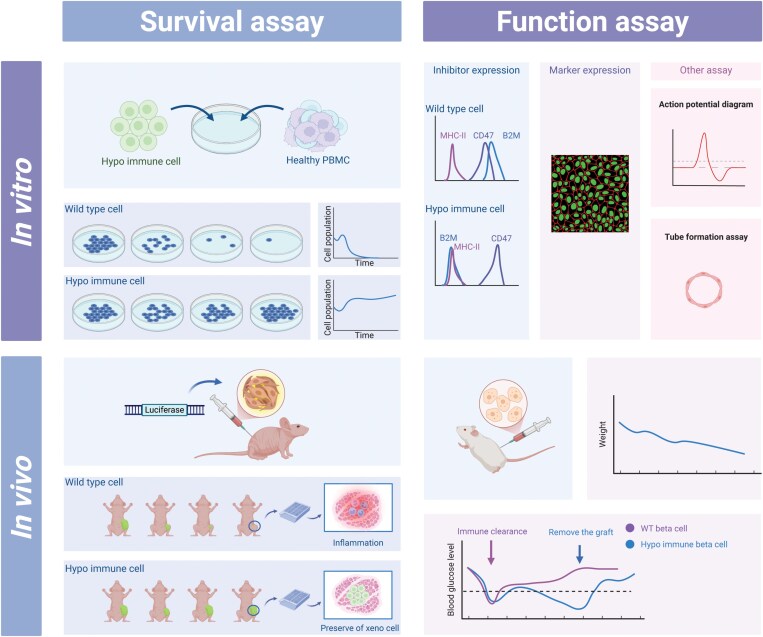
Methods for assessing immune privileged cells.

Current models are unitary, including *in vitro* mixture with immune cells or injected into humanized mice (Fig. 4). The assessment only focuses on the survival rate of the transplanted cells, mostly PSC, some tried differentiated cells. Yet we lack the evaluation of the function of these derivatives. It should be testified that the immune privileged strategies are compatible with exercising its original function. Islet or pancreatic beta cell transplantation has taken the lead in related fields. Yoshihara et al. clarified that the islet-like organoids are effective in producing insulin and lowering blood glucose levels in immune-competent mice for 50 days [41]. Feng et al. detected the electrophysiological activities of human iPSC-derived neural progenitor cells after they were transplanted into the monkey forebrain, indicating that the cells matured and functioned robustly [84]. Researchers are gradually breaking through bottlenecks to achieve longer survival and better function.

Differentiation and its assessment are the obstacles in the whole field of regenerative medicine, particularly for immune tolerance. Some typical biomarkers are used as evaluation criteria in differentiation, like CD31 for endothelial cells, cTnT for cardiomyocytes. Senior standards focus on functionality.

### Limitation in PSC

iPSC generation and quality control are the main obstacles in regenerative medicine commercialization. Multiple strategies of iPSC generation are under evaluation to make sure that genome contamination is avoided [85]. Sendai virus might be a good choice because it delivers RNA with no random insertion of exogenous sequences into the host genome. Yet this approach is too expensive for widespread application. Moreover, the engineering strategies to induce immune tolerance only exacerbate the issue with the complicated editing, selecting, and verifying processes. Besides, the wide application of universal PSC is based on the guarantee of mature and promising differentiation protocols to various target cell lines. Unfortunately, we still lack experimental proof of directed differentiation, such as HSC lineage, even irrespective of immune privilege. As regards immune tolerance, some researches successfully generated hipo-immune PSC-derived endothelial cells, cardiomyocytes, pancreatic beta cells, and islet-like organoids [18, 21, 41, 44]. More are exploring primary cells [19, 86]. Another target cell in immune tolerance is CAR-T. As a novel cancer therapy, CAR-T cells precisely recognize and kill tumor cells. To prevent the clearance from the host immune system, applying an immune tolerance strategy is effective in maintaining the CAR-T population [19].

## Conclusion

Immune tolerance appears to be the last barrier in turning regenerative medicine into a truth. As an optimal solution, the generation of hypo-immune cells encounters practical difficulties. Briefly, researchers get inspired by the immune process and tumor escape to propose various strategies to help the allograft or xenograft evade the host immune attack. HLA cloak has been the core issue but the “missing self” recognition blocks the way. The compromising approach of HLA selective depletion has proved to be effective yet reduces the generality. The joining of immune blockades overexpression pushes the progress of allograft survival while it makes the whole scheme more and more complicated. Besides, the time and financial cost of gene manipulation will become a problem in clinical applications. As for co-transplantation strategies, more reliable research is needed to confirm its effectiveness. The side effects of overexpressing immune blockades and adding extra cells still need confirmation before entering clinical trials.

Although the field represents an exciting research avenue, numerous challenges and uncertainties persist. For instance, ensuring the long-term survival and functionality of transplants, require further investigation and exploration. Integrated application of immune manipulation and isolation material conjointly energizes the field [87]. Consequently, additional basic research endeavors and clinical trials are imperative to validate the safety and efficacy of immune tolerance in iPSCs transplantation.
